# Nanoscopic Spatial Association between Ras and Phosphatidylserine on the Cell Membrane Studied with Multicolor Super Resolution Microscopy

**DOI:** 10.3390/biom12081033

**Published:** 2022-07-26

**Authors:** Anna M. Koester, Kai Tao, Malwina Szczepaniak, Matthew J. Rames, Xiaolin Nan

**Affiliations:** 1 Program in Quantitative and Systems Biology, Department of Biomedical Engineering, Oregon Health & Science University, 2730 S Moody Ave., Portland, OR 97201, USA; koestera@ohsu.edu (A.M.K.); taok@ohsu.edu (K.T.); szczepam@ohsu.edu (M.S.); 2 Cancer Early Detection Advanced Research Center, Knight Cancer Institute, Oregon Health & Science University, 2720 S Moody Ave., Portland, OR 97201, USA; rames@ohsu.edu

**Keywords:** Ras GTPases, membrane nanodomains, nanoclusters, Ras dimers, phosphatidylserine, super resolution microscopy, DNA-PAINT, colocalization analysis, actin cytoskeleton

## Abstract

Recent work suggests that Ras small GTPases interact with the anionic lipid phosphatidylserine (PS) in an isoform-specific manner, with direct implications for their biological functions. Studies on PS-Ras associations in cells, however, have relied on immuno-EM imaging of membrane sheets. To study their spatial relationships in intact cells, we have combined the use of Lact-C2-GFP, a biosensor for PS, with multicolor super resolution imaging based on DNA-PAINT. At ~20 nm spatial resolution, the resulting super resolution images clearly show the nonuniform molecular distribution of PS on the cell membrane and its co-enrichment with caveolae, as well as with unidentified membrane structures. Two-color imaging followed by spatial analysis shows that KRas-G12D and HRas-G12V both co-enrich with PS in model U2OS cells, confirming previous observations, yet exhibit clear differences in their association patterns. Whereas HRas-G12V is almost always co-enriched with PS, KRas-G12D is strongly co-enriched with PS in about half of the cells, with the other half exhibiting a more moderate association. In addition, perturbations to the actin cytoskeleton differentially impact PS association with the two Ras isoforms. These results suggest that PS-Ras association is context-dependent and demonstrate the utility of multiplexed super resolution imaging in defining the complex interplay between Ras and the membrane.

## 1. Introduction

The inner membrane Ras small GTPases function as molecular switches in canonical signaling pathways by cycling between an active GTP-bound and an inactive GDP-bound state, and in so doing regulate cell growth, proliferation, and metabolism [1,2]. As key regulators of cell physiology, Ras activities are carefully tuned in normal cells, and loss of this regulation, such as through mutations, is frequently associated with diseases such as human cancers [3].

It has been well established that in addition to GTP loading, Ras molecules have to be positioned on the membrane in order to be biologically active [4,5]. Membrane targeting of Ras is achieved primarily through the C-terminal hypervariable region (HVR), which differs substantially in both the amino acid sequence and the post-translational modifications among the three main Ras isoforms H-, K-, and NRas [6,7]. Heterogeneity in HVR post-translational modification leads to isoform-specific enrichment of Ras in spatially and compositionally distinct nanoscopic membrane domains, where Ras molecules aggregate to form multimers, including nanoclusters and dimers [8,9,10,11]. Recent work by Zhou et al. has shown colocalization of different lipid species in the nanoclusters of multiple Ras isoforms [12]. The Ras G-domain, which is homologous among the isoforms, interacts with lipids [13,14,15,16]. In line with this, recent biochemical studies and large-scale molecular dynamics simulations suggest that the exact lipid composition of the membrane likely dictates both Ras–Ras and Ras–effector interactions [15,16,17,18,19,20,21]. Of these lipids, PS is a shared component between H- and KRas nanoclusters, suggesting its potential involvement in the spatial organization and function of Ras on the cell membrane [22,23,24,25].

In healthy mammalian cells, PS localizes to the inner leaflet of the plasma membrane, making up 20% of all membrane lipids [26,27]. Its high abundance considerably influences the overall negative charge density of the membrane as well as the distribution of proteins with cation stretches, such as KRas [27]. Aside from the well-known electrostatic interactions of its poly-lysine stretch with PS, the KRas HVR encodes molecular specificity for PS [28]. PS depletion results in mislocalization of KRas and reduced nanoclustering [23,29], whereas enhanced clustering of PS enhances KRas-G12V nanoclustering and signaling [30]. These studies highlight the need to understand Ras function in the context of membrane organization and lipid composition. 

Previous efforts to map the intracellular distribution of PS culminated in the discovery of Lact-C2-GFP as a specific PS sensor [27]. Using the same Lact-C2-GFP sensor, Yeung et al. were able to visualize the cytoplasmic-facing distribution of PS and its ability to recruit charge-based proteins to the membrane [27]. High resolution mapping using immuno-EM imaging of BHK cell membrane sheets in combination with expression of Lact-C2-GFP showed that PS forms clusters at the inner leaflet of the membrane, is associated with caveolae, and co-segregates with cholesterol [31]. Single particle tracking has revealed different pools of PS in the cell membrane that respond to specific perturbations [26]. To date, however, it remains to be seen whether PS distribution and its relationship with Ras in intact cells are consistent with these observations.

The recent advent of super resolution microscopy (SRM) has allowed spatial mapping of biological molecules within intact cells with 10–20 nm lateral resolution [32,33,34,35]. SRM has been successfully used to study dimerization, multimerization, and diffusion of Ras proteins in the membrane [10,11]. New additions to the toolkit, such as DNA point accumulation in nanoscale topography (DNA-PAINT) [34], have greatly simplified multicolor SRM into multiple cycles of single-color imaging. In each imaging cycle, a short fluorophore-conjugated imaging strand (IS) oligonucleotide transiently binds to a complementary docking strand (DS) oligonucleotide attached to the target of interest to yield short-lived ‘blinking’ (localization) events. These single-molecule events are then localized with sub-diffractive precision, typically on the order of 8–10 nm [36,37], which reports the positions of the underlying targets. A super-resolved image of the target can then be derived from the summation of these precise localizations. We have recently introduced an improved DNA-PAINT called DNA-PAINT-ERS [36] which has enhanced localization kinetics, improved overall image quality, and increased imaging speed, which are particularly useful features for multiplexed SRM imaging. These advanced imaging tools could complement immuno-EM and help build a more complete view of Ras and its associated membrane compartments, including both lipids and proteins.

Here, we report dual-color SRM imaging of both PS and Ras (HRas-G12V and KRas-G12D) at nanoscale resolution in intact cells and the analysis of their spatial associations. Using the sensor Lact-C2-GFP in combination with DNA-PAINT, we obtained images showing the molecular distribution of PS. SRM images of PS showed that it is universally expressed across the entirety of the cell membrane and is nonuniformly distributed at the ~100 nm scale or below. Two-color DNA-PAINT-ERS imaging of both PS and Ras followed by spatial analysis showed that PS co-organizes with both KRas and HRas. HRas seems to have a stronger co-organization with PS than KRas. Perturbation of the actin cytoskeleton suggests actin involvement in the regulation of PS–Ras interactions. Our results demonstrate the utility of multiplexed super resolution imaging in defining the complex interplay between Ras and PS, and complement past studies by showing that PS–Ras association is context-dependent (including isoform).

## 2. Materials and Methods

### 2.1. Materials

All cell culture reagents and supplies were purchased from Thermo Fisher Scientific (Waltham, MA, USA) unless otherwise specified. Primers and DNA oligonucleotides were synthesized by Integrated DNA Technologies (Coralville, IA, USA).

### 2.2. Plasmids 

Lact-C2-GFP (#22852) was a gift from Dr. Sergio Grinstein (Addgene, Watertown, MA, USA) [27] and pGEX6P1-GFP-Nanobody (#61838) was a gift from Dr. Kazuhisa Nakayama (Addgene, Watertown, MA, USA) [38]. The pGEX6P1-GFP-nanobody plasmid was modified by adding an amber codon (TAG) and a 6 × His-tag sequence (5′-CACCACCACCACCACCAC-3′) to the C-terminal end of the GFP open reading frame. Primers were designed to clone the His-tagged anti-GFP-nanobody sequence into a pET21b vector backbone (Table 1). First, Hi-Fidelity PCR was performed to linearize the anti-GFP-nanobody insert and pET21b vector backbone. Afterwards, PCR amplification insert and backbone were assembled using In-Fusion HD cloning technology (#638910) according to the manufacturer’s instructions (Clontech, Mountain View, CA, USA).

### 2.3. Nanobody Purification and Conjugation with DNA Oligos

The His-tagged pET21b-GFP-nanobody plasmid was transformed into *Escherichia coli* BL21 (#C600003) and protein expression was induced by addition of 1 mM IPTG (#15529019) to exponential-phase bacteria (OD600 = 0.5). After 5 h at 30 °C with constant shaking, the bacteria were harvested by centrifugation (10 min, 10,000× *g*, 4 °C). During the following procedures, cells and protein solutions were kept at 4 °C at all times to avoid protein degradation. Bacteria were lysed by sonication (4 × 30 s bursts at 300 W with 30 s cooling periods) in lysis buffer (46.75 mM Na_2_HPO_4_, 3.25 mM NaH_2_PO_4_, 300 mM NaCl, 10 mM imidazole, pH 8.0) on ice. After centrifugation (30 min, 13,000× *g*), a clear lysate was obtained by collecting the supernatant. The protein lysate was incubated with Ni-NTA Agarose (#30210 Qiagen, Hilden, Germany) with mechanical rotation for 2 h. The lysate–Ni-NTA mixture was transferred into a column and washed with wash buffer (46.75 mM Na_2_HPO_4_, 3.25 mM NaH_2_PO_4_, 300 mM NaCl, 20 mM imidazole, pH 8.0). Protein was eluted by centrifugation with elution buffer (46.75 mM Na_2_HPO_4_, 3.25 mM NaH_2_PO_4_, 300 mM NaCl, 300 mM imidazole, pH 8.0). To remove excess imidazole, desalting was performed using size exclusion chromatography on an ÄKTA Pure system (GE Healthcare, Chicago, IL, USA) with a 5 mL Cytiva HiTrap HP desalting prepacked column (#GE17-1408-01 Sigma, St. Louis, MO, USA).

Anti-GFP-nanobodies were conjugated to docking strand (DS) oligos via dibenzocyclooctyne (DBCO)-azide click chemistry. DS sequences contained a 5′ amino modifier C6 and a 3′ Cy3 fluorophore. To first conjugate DS with DBCO-PEG4-NHS ester, the latter was added to DNA in 20 × molar excess. A 50 μL reaction volume of DBCO-ester, DS, and ultra-pure water with pH adjusted to 8.5 using sodium bicarbonate was incubated on a shaker for 3 h at room temperature. Ethanol precipitation was carried out twice with 0.3 M sodium acetate at −80 °C to purify the DS-DBCO product. The DS-DBCO product was then reacted in 10 × molar excess with the azide groups of the anti-GFP–nanobody via copper-free click chemistry overnight on a shaker at room temperature. The final anti-GFP-nanobody-DS product was purified using a HiTrap Q HP anion exchange chromatography column (#GE17-1153-01 Sigma, St. Louis, MO, USA) with an ÄKTA Pure system.

The resulting product was applied to 10 kDa Amicon^®^ Ultra Columns (#ACS501024 Millipore, St. Louis, MO, USA) to remove excess unconjugated nanobodies. The final anti-GFP-nanobody-DS was resuspended in PBS and stored at −20 °C until sample labeling was carried out. Intermediate products were run on Bris-Tris gradient gels (4–12%, #NP0323) and stained with InstantBlue^®^ Coomassie Protein Stain (#ab119211 Abcam, Cambridge, UK).

### 2.4. Cell Culture and Transfection

Halo-tagged HRas-G12V and SNAP-tagged KRas-G12D coding sequences were placed under a CMV promoter regulated by the TetOn operon. Both constructs were transduced into an isogenic U2OS-tetR cell line that constitutively expressed the tet repressor (tetR) derived from the parent U2OS (HTB-96 ATCC, Manassas, VA, USA) using lentivirus. Single-cell clones expressing HRas-G12V and KRas-G12D fusion proteins under doxycycline regulation were isolated and used for experiments. Cells were maintained in Gibco DMEM (#11995073) supplemented with 10% FBS (#26140079) at 37 °C and 5% CO_2_, passaged every 3–4 days, and used below passage 20. Prior to experiments, cells were plated in eight-well Lab-Tek II chambers (#155360). To induce expression of HRas-G12V and KRas-G12D, cells were exposed to 50 ng/mL doxycycline (#17086-28-1 Sigma, St. Louis, MO, USA) for 48 h. Cells were transfected with 200 ng Lact-C2-GFP plasmid and Lipofectamine 3000 transfection reagent (#L3000015) according to the manufacturer’s instructions in OptiMEM reduced serum medium (#31985062) for 5–6 h. Afterwards, the transfection reagent was replaced with fresh DMEM supplemented with 10% FBS and 50 ng/mL doxycycline. At 24 h after transfection, Lact-C2-GFP was expressed at desired levels and cells were treated with either 2 μM Latrunculin A (#L5163 Sigma, St. Louis, MO, USA) for 10 min or 15 μM Jasplakinolide (#420127 Sigma, St. Louis, MO, USA) for 30 min at 37 °C. Subsequently, cells were fixed with 3.7% paraformaldehyde in 1 × PHEM buffer (60 mM PIPES, 25 mM HEPES, 10 mM EGTA, and 4 mM MgSO_4_, pH 7.0).

### 2.5. Immunostaining

After fixation, cells were quenched with 300 mM glycine (Sigma #W328712) and permeabilized with 0.2% saponin (#S7900 Sigma, St. Louis, MO, USA) in PBS for 40 min at room temperature. Cells were incubated with Image-iT™ FX Signal Enhancer (#I36933) for 40 min in the dark before blocking with 2% bovine serum albumin (#A9418 Sigma, St. Louis, MO, USA) supplemented with 5% salmon sperm DNA (#15632011) in PBS for 40 min. Subsequently, cells were incubated with anti-GFP-nanobody-DS to stain PS and either SNAP- or Halo-tag-substrate-DS to stain KRas and HRas, respectively, in 2% BSA supplemented with 5% salmon sperm DNA in PBS for 2 h on a rocker in the dark. Cells were post-fixed with 3.7% PFA supplemented with 0.2% glutaraldehyde.

### 2.6. Microscopy

All SRM data were obtained with a custom-built single molecule imaging system. In brief, lasers emitting at wavelengths of 488 nm, 561 nm, and 647 nm were combined and introduced into the back of a Nikon Ti-U microscope. An f = 400 mm lens was used to focus the collimated laser light to the back aperture of a 60X TIRF objective to achieve total internal reflection illumination of the sample. Images were acquired with strict TIRF illumination to probe membrane proteins appropriately. During data acquisition a custom-built focus stabilization system based on the detection of the reflected excitation laser was used. A multi-edge polychroic mirror and emission filters FF01-525/45, FF01-605/64, and FF01-708/75 (Semrock, Rochester, NY, USA) were used to reflect laser light into the objective and clean up fluorescent signals. Images were collected using an iXon Ultra electron-multiplied charge coupled device (Andor, Abingdon, UK) with an EM gain of 200 and 30–40 ms exposure time. The power density of the 647 nm laser for DNA-PAINT(ERS) imaging was typically ~500 W cm^−2^.

### 2.7. Data Acquisition and Analysis

All images were acquired using the micromanager software suite v1.4 (San Francisco, CA, USA) (https://micro-manager.org/ accessed on 10 February 2020) and saved as TIF files. For HRas and KRas, targets of 60,000–80,000 frames were acquired. For PS, we generally acquired 120,000–160,000 raw image frames, corresponding to an average of 5 × 10^6^ localizations. In-house Matlab scripts were used for single-molecule localization, typically at a spatial resolution of ~20 nm, as well as for localization filtering, sorting, and rendering for visualization [39]. In brief, drift correction was performed using 50 nm gold nanoparticles (#J67157.AC) as fiducials; raw localizations were filtered based on signal to noise ratio, width of point spread function, and aspect ratio followed by sorting, during which events that appeared within a defined number of frames (typically two) and distance (typically 80 nm) were combined into a single event with averaged coordinates. The sorted localizations for both channels were saved as .cor files and then registered with an affine transformation matrix using in-house Matlab scripts. Coordinate files were converted into .csv format and loaded into Coloc-Tesseler software v1.0 (https://github.com/flevet/Coloc-Tesseler/releases/tag/v1.0) (Bordeaux, France) [40]. Analysis of co-organization of PS and Ras was performed on molecule coordinates as directed in the software manual, and the plots and graphs were prepared using Prism software v9 (Graphpad, San Diego, CA, USA).

## 3. Results

### 3.1. Phosphatidylserine Plasma Membrane Distribution at the Nanoscale

**Figure 1:**
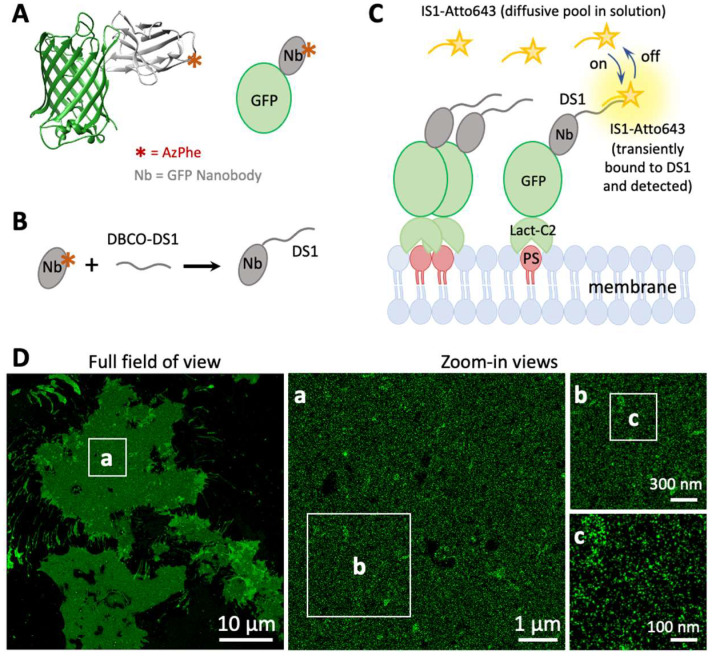
Super resolution imaging of plasma membrane PS in U2OS cells using a combination of the Lact-C2-GFP sensor with DNA-PAINT. (**A**) Structure of the GFP-enhancer nanobody (GFP-Nb) complex (based on PDB ID 3K1K). The location of the artificial amino acid (AzPhe) is indicated by a red asterisk. (**B**) Schematic for the conjugation of the GFP-Nb with a single-stranded DNA oligonucleotide via site-specific click chemistry between AzPhe and dibenzocyclooctyne (DBCO); shown here is the reaction with DBCO-modified docking strand 1 (DS1). (**C**) Schematic of DNA-PAINT imaging showing complementary imager strands (IS1) diffusing above the sample. Binding of IS1 (originally in solution) to DS1 (immobilized on target) and subsequent unbinding generate transient (~0.1 s) single-molecule signals, which accumulate over time to probe all DS1 on the sample. (**D**) Example super resolution image of PS in U2OS cells from the DNA-PAINT process depicted in (**C**). Lact-C2-GFP was transiently expressed in U2OS cells and labeled with the Nb-DS1 prior to imaging. The imaging was performed under total internal reflection to limit the detection volume to the ventral membrane. The image on the left is an overview, with serial magnifications shown in (**a**–**c**).

We combined the use of the Lact-C2-GFP biosensor with DNA-PAINT(ERS) imaging to visualize PS at the nanoscale in intact cells. For the detection of Lact-C2-GFP, we used an anti-GFP nanobody (GFP-Nb) known as the ‘GFP-enhancer’ (Figure 1A) [41], a much smaller (~15 kDa) affinity agent compared with full length anti-GFP antibodies (~150 kDa). Probing GFP with the miniature GFP-Nb will allow efficient detection of PS, reduce the linkage error (Figure 1A), and help improve the achievable spatial resolution [42]. 

**Figure A1:**
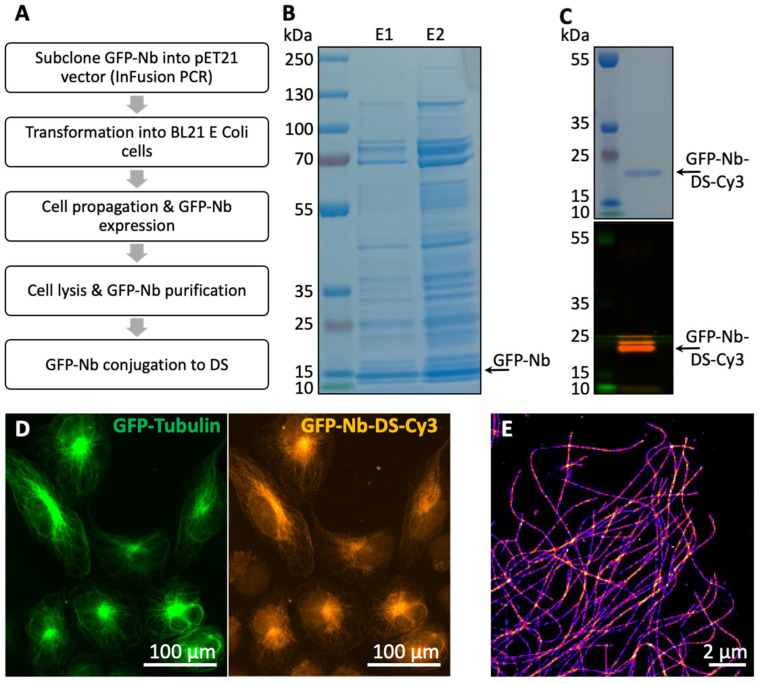
Anti-GFP-nanobody purification and conjugation with DNA oligos. (**A**) Schematic of GFP-Nb purification workflow. (**B**) SDS-PAGE (NuPAGE Bis-Tris 4–12%) gel of GFP-Nb purified by Ni-chelating chromatography after Coomassie staining. The GFP-Nb was detected at an expected band size of 15 kDa. E1 = first elution and E2 = second elution. (**C**) SDS-PAGE (NuPAGE Bis-Tris 4–12%) gel of purified GFP-Nb after conjugation to DS labeled with Cy3 via clicking of AzPhe after Coomassie staining (top) and Cy3 in-gel fluorescence (bottom) detected by excitation with green light and filter F595 (for 595 nm). (**D**) Fluorescence images of U2OS cells transiently transfected with GFP-tubulin (green) labeled with GFP-Nb conjugated to DS1-Cy3 (yellow). (**E**) DNA-PAINT image of U2OS cells expressing GFP-tubulin stained with GFP-Nb.

For DNA-PAINT imaging, the GFP-Nb is conjugated to a single-stranded DNA oligo designated as the docking strand (DS, specifically DS1 in this case) at the artificial amino acid 4-azido-phenylalanine (AzPhe) via a copper-free click reaction (Figure 1B and Figure A1). The AzPhe is located at the C-terminal of the GFP-nanobody (position 116) to ensure that the attached DS1 does not interfere with the GFP-Nb domains required for GFP binding. The specificity of the DS1-modified GFP-Nb for DNA-PAINT imaging was confirmed by imaging microtubules in U2OS cells (Figure A1D–E) and by the complete lack of localization signals in the non-transfected cells.

Using this strategy, we studied the distribution of PS in the plasma membrane of U2OS cells. The imaging was confined to the membrane and the immediate cytoplasmic space by illuminating the sample under strict total internal reflection (TIR). Figure 1D shows representative super resolution images of PS acquired by imaging Lact-C2-GFP transiently transfected into the cells. At the whole cell level, PS expression appeared to be largely uniform across the entire bottom membrane, with no density gradient at first sight. Stronger staining of Lact-C2-GFP was observed at the cell periphery where the membrane folds back. On the edges of the cells where filopodia are formed, PS molecules appeared as bright accumulations, possibly due to overlapping membranes (Figure 1D). This observation is consistent with results from previous low-resolution imaging studies showing universal expression of PS on the inner leaflet of the entire cell membrane [26,27]. 

Compared with the apparent diffusive distribution of PS across the whole bottom membrane, zoom-in views of the super-resolved images show a more heterogenous molecular distribution of PS. Between the sub-µm to nanoscales (Figure 1D(a,b)), small areas of PS enrichment between 50–200 nm in diameter as well as larger areas between 500–700 nm in diameter could be readily observed. As shown in the next section, a subset of these PS-enriched structures with diameters between 50–200 nm are caveolae; the identity of the other structures is currently unknown. Further zoom-ins at the ~100 nm scale (Figure 1D(c)) revealed a multitude of dense assemblies of PS (~20–100 nm in diameter) interspersed with lower density signals, and regions completely devoid of PS were rare. These observations are largely consistent with previous high-resolution imaging studies on membrane sheets [31].

### 3.2. Imaging the Spatial Association between KRas-G12D and PS at the Nanoscale

**Figure 2:**
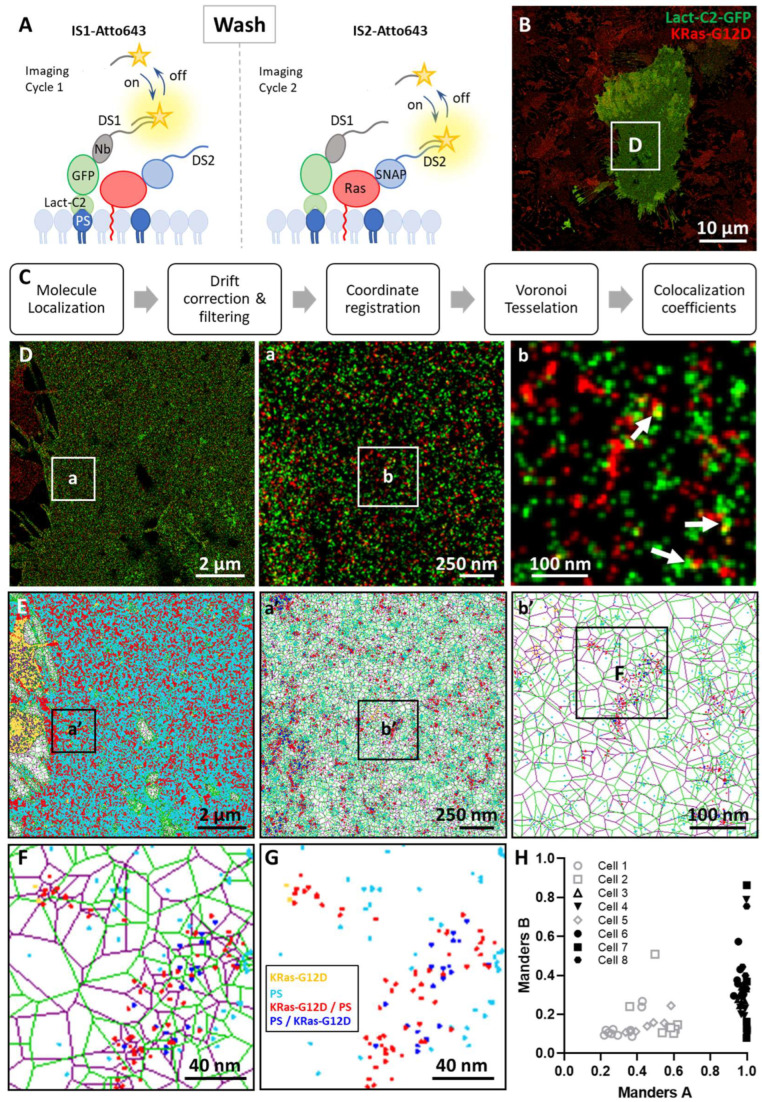
Multiplexed imaging of PS and KRas-G12D using exchange-PAINT and assessment of colocalization with Coloc-Tesseler. (**A**) Schematic of dual-color imaging with fluorescently conjugated imaging strands, each targeting the corresponding docking strand. DS1 conjugated to the GFP-nanobody (Nb-DS1, grey) binds to the transiently transfected PS sensor Lact-C2-GFP (green) and is recognized by IS1-Atto643. DS2 conjugated to the SNAP-substrate reacts with SNAP tag to form a covalent linkage and is recognized by IS2. The two targets are probed sequentially in two imaging cycles with a washing step in between to remove the imager strand used in the previous cycle, allowing the same (bright) fluorophore to be used for imaging both targets. (**B**) Example dual-color DNA-PAINT image of PS (green) and KRas-G12D (red) in U2OS cells. All cells in the field of view expressed SNAP-KRas-G12D, and only the cell in the middle was transiently transfected with Lact-C2-GFP. (**C**) Flow chart for the image analysis. (**D**) Magnified region from (**B**) with further magnified areas (**a**) and (**b**) showing distinct molecular distribution of PS (green) and KRas-G12D (red). (**E**) Colocalization analysis of magnified areas namely (**a’**) and (**b’**) that correspond to the same regions shown in (**a**) and (**b**) of region (**D**) using Coloc-Tesseler, showing the Voronoi diagrams for PS (green mesh) and KRas-G12D (purple mesh). Regions of molecular enrichments are shown as dots in distinct colors. (**F**) Magnification of the Voronoi diagram of PS (green) and KRas-G12D (purple), including classification of molecule coordinates into four orthogonal classes dependent on their local pair-normalized localization densities (cyan = PS enriched alone; yellow = KRas-G12D enriched alone; red = KRas-G12D co-enriched with PS; and blue = PS co-enriched with KRas-G12D). Non-enriched localizations in the PS or KRas channels are not shown. (**G**) Molecule coordinates divided into the four orthogonal classes without Voronoi diagrams. (**H**) Plot of Manders A (fraction of KRas-G12D co-enriched with PS of all enriched KRas-G12D, or # of red dots/# of red plus yellow dots) versus Manders B (fraction of PS co-enriched with KRas-G12D of all enriched PS, or # of blue dots/# of blue plus cyan dots) coefficients based on the classifications shown in (**E**–**G**). The experiment was performed in triplicate (N = 3) and included eight cells overall, with 55 analyzed regions of interest (ROIs).

We next aimed to study the spatial relationship between PS and KRas-G12D. In this work we focused on mutants of Ras that are constitutively active and drive signaling. We genetically fused the SNAP tag to the N-terminus of KRas-G12D, which allowed labeling of single KRas proteins 1:1 with a DNA-PAINT DS conjugated to a SNAP substrate (O6-benzylguanine or BG). The SNAP tag is similar in size to GFP, thus offering a similar advantage to using the GFP/GFP-Nb strategy, as in the case of PS labeling (Figure 2A). U2OS cells stably expressing SNAP-tagged KRas-G12D protein under doxycycline regulation were transiently transfected with Lact-C2-GFP. SNAP-KRas-G12D and PS (Lact-C2-GFP) were then probed through two-cycle imaging using the two orthogonal DS-IS pairs, namely, DS1-IS1 (PS) and DS2-IS2 (KRas-G12D), in a strategy known as exchange-PAINT [34,43]. Specifically, after imaging Lact-C2-GFP in the first round, IS1 was removed through a simple washing step (as IS1 only transiently binds to DS1), and IS2 was then added for imaging KRas (Figure 2A).

Figure 2B shows the molecular distribution of both PS and KRas-G12D in a typical cell obtained with two-color DNA-PAINT. As expected, KRas-G12D was universally detected across the plasma membranes of all cells in the sample, whereas PS was detected in a smaller fraction of cells transfected with Lact-C2-GFP (Figure 2B). In the zoom-in views it appeared that PS and KRas-G12D co-occur in similar membrane regions and are both absent from others (Figure 2D(a)). The co-occurrence of the two molecules is more evident when both targets were present in higher densities (indicated by the arrows in Figure 2D(b)). This suggests a potential spatial relationship between PS and KRas-G12D. However, direct, pixel-to-pixel overlaps between PS (green) and KRas-G12D (red) were rare (Figure 2D(b)). 

We note that this lack of pixel-to-pixel overlap is typical in SRM and EM images of supposedly ‘colocalizing’ targets [24,44]. In conventional diffraction-limited fluorescence microscopy, potential interaction of biological targets is often evaluated through assessment of simple spatial overlap at the pixel level. Colocalization analysis of single-molecule localization microscopy (SMLM) images is more challenging because the images contain millions of molecule coordinates and co-organization cannot be assessed as pixel-overlay, only as co-occurrence of localizations. This challenge has only now started to be addressed, with a recent addition to the toolbox being Coloc-Tesseler [40]. 

Coloc-Tesseler analyzes ‘colocalizations’ in SMLM images as spatial associations between molecular targets (Figure 2C). This is achieved by first creating a Voronoï diagram, which is a set of polygonal regions around each localization [45]. Each polygon is the collection of all points nearest to the ‘seed’ localization (molecular coordinate). The geometric traits of each polygon for the instance area and shape provide information about the neighborhood surrounding each molecule and allow computation of a variety of parameters that provide quantitative information (Figure 2E). To briefly explore the origin of certain circular or elongated membrane structures that were found to be enriched in PS (Figure 2D,E), we performed co-staining for caveolae followed by Coloc-Tesseler analysis. This showed significant overlap between the two targets (Figure A3), as has been previously reported [31]. 

Coloc-Tesseler computes the normalized density of the immediate (first rank) neighbor polygons based on the PS (green) and the KRas-G12D (purple) Voronoï diagram and compares this to the average density of a reference distribution of spatial randomness for each channel independently (Figure 2F). Automatic thresholding of both channels represented by the different colors allows for the classification of molecules into a high-density class for each channel (cyan = PS and yellow = KRas-G12D) and one low-density background class (black; not shown). High density areas of both channels are further subdivided into two classes depending on whether or not a molecule of a given channel lies inside a high-density area of the other channel (blue = PS in KRas-G12D and red = KRas-G12D in PS). 

As such, PS and KRas-G12D localizations are organized into four different orthogonal classes (Figure 2F,G) that indicate their co-organization based on their local pair-normalized localization densities. Independent and parameter-free classification of PS and KRas-G12D into these classes allows computation of the Manders overlap coefficient, which reliably quantifies the levels of colocalization independent of molecule densities of KRas-G12D or PS. Two Manders coefficients were computed: Manders A indicating KRas-G12D, associated with PS, and Manders B, indicating PS, associated with KRas-G12D (Figure 2G).

**Figure A2:**
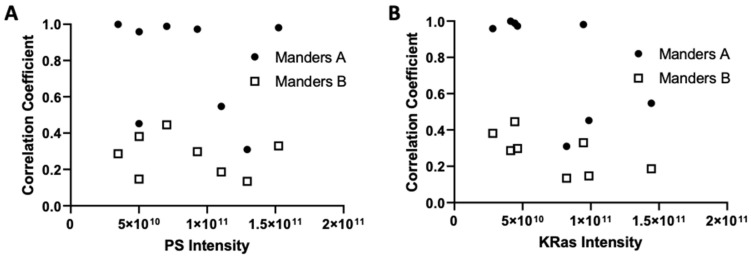
Independence of DNA-PAINT image signal intensity measured using Image J and colocalization coefficients obtained from Coloc-Tesseler. (**A**) The scatterplot shows the whole cell intensity of the PS channel normalized to area plotted against both the Manders A (circles) and B (squares) colocalization coefficients of eight KRas-G12D U2OS cells with PS (each symbol represents one cell in the plot). (**B**) The scatterplot shows the whole cell intensity of the KRas channel normalized and plotted in the same way. Both plots show no relationship between intensity levels of either PS or KRas, and colocalization coefficients describing their co-organization, which rules out that increased co-organization is simply due to higher levels of probes.

**Figure A3:**
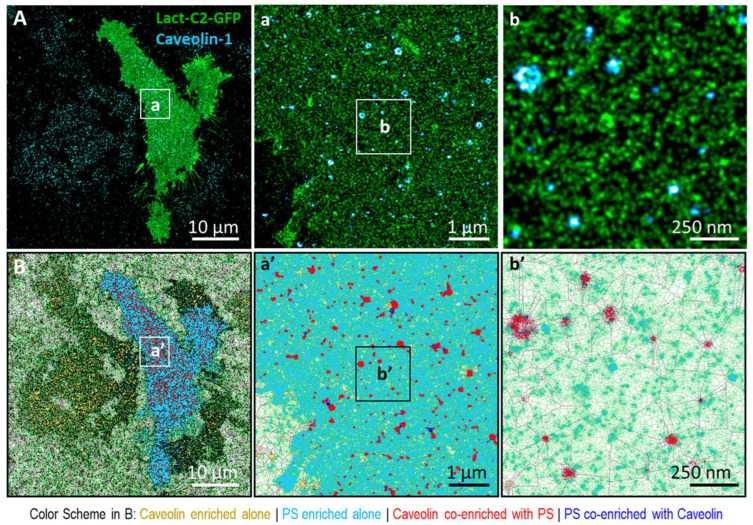
Multiplexed imaging of PS and caveolae using exchange-PAINT and qualitative assessment of co-organization with Coloc Tesseler. (**A**) Single DNA-PAINT image of PS and caveolae in U2OS cells and magnified regions from (**A**) namely (**a**) and (**b**) showing the distinct molecular distribution of PS (green) and caveolae (cyan). (**B**) Colocalization analysis with Coloc-Tesseler of region (**A**) with the same magnified areas namely (**a’**) and (**b’**) showing the Voronoi diagram for PS (blue) and caveolae (yellow). Magnifications show classification of molecule coordinates into five orthogonal classes dependent on their local pair-normalized localization densities (cyan = dense PS; yellow = dense caveolae; red = caveolae overlap with PS; blue = PS overlap with caveolae). It can be seen that caveolae directly co-organize with patches of PS (indicated by the red color).

Figure 2H summarizes the results we obtained from regions of interest (ROI). The Manders A coefficient (KRas-G12D co-enriched with PS) of the ROIs varied between 0.2–0.6 (weak to moderate association) and 1 (complete association). Within one cell, the variations in both coefficients varied to a much lesser extent. By plotting Manders A against Manders B, it appeared that there were two populations of cells in terms of the association of KRas-G12D with PS (Figure 2H). In half of the cells, KRas-G12D colocalizes with PS at nearly 100%, whereas in the other half about 50% of KRas-G12D molecules are co-organized with PS molecules reflected by the two colors in the scatterplot. The difference between the two populations is not due to their differences in the amount of detected PS (e.g., due to the transient transfection protocol) nor to the expression level of KRas-G12D (see Figure A2). The Manders B coefficient, which quantifies the fraction of PS co-enriched with KRas-G12D, was much lower in the same ROIs. On average, only 26% of PS sampled in our experiments were co-organized in similar membrane compartments as KRas indicated by the Manders B coefficient. As would be expected, PS is much more abundant even though it is likely to be sampled at a lower rate (i.e., detected PS vs. actual amount of PS) than KRas G12D, and should co-enrich with both KRas-G12D and many other targets, hence the much lower Manders B coefficients. 

### 3.3. Imaging and Quantitating Interaction between HRas-G12V and PS

Previous immuno-EM studies have suggested that PS is a component of both HRas and KRas nanoclusters. Ras isoforms interact with the membrane in an isoform-specific manner, which applies to Ras-PS interactions as well, and it remains to be seen how the two Ras isoforms interact and/or spatially associate with PS in cells. 

We therefore studied the co-organization of HRas-G12V and PS using a similar imaging strategy. We genetically fused HRas-G12V to the Halo-tag and stably expressed the fusion protein in U2OS cells followed by transient transfection with Lact-C2-GFP, as previously described. The Halo-tag was reacted with a chloroalkane linker substrate conjugated to DS3 to perform dual-color exchange-PAINT imaging, with Halo-HRas-G12V imaged using the corresponding imager strand IS3.

**Figure 3:**
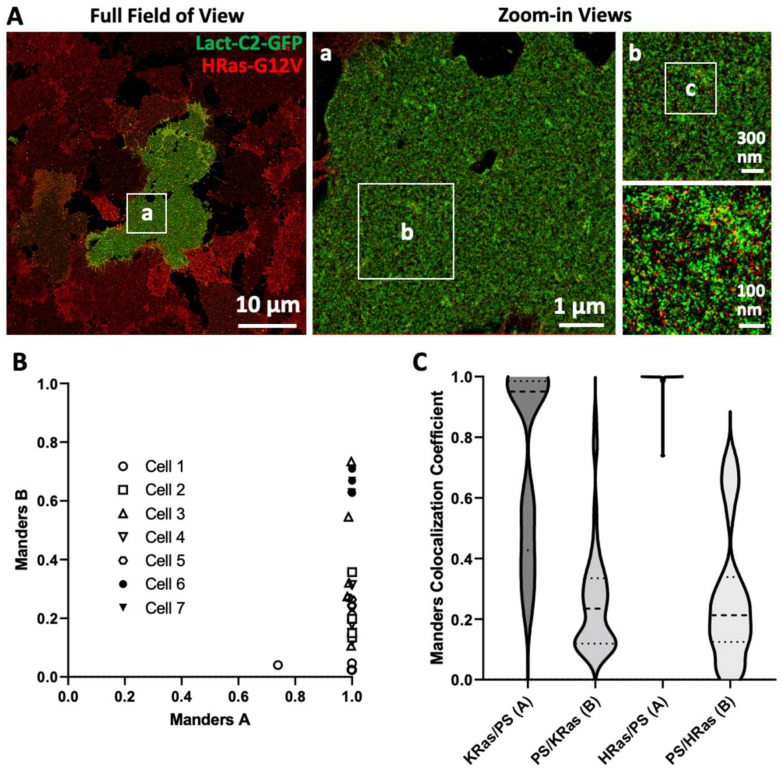
Dual color super resolution imaging of PS and HRas-G12V using exchange-PAINT and comparison of the spatial associations between PS and HRas-G12V or KRas-G12D. (**A**) Example dual-color DNA-PAINT image of transiently transfected Lact-C2-GFP (bound to PS) and stably expressed Halo-HRas-G12V in U2OS cells. Halo-HRas-G12V was imaged using a similar strategy to that for SNAP-KRas-G12D shown in Figure 2. Magnified views (**a**–**c**) show the molecular distributions of both targets. (**B**) Quantitation of Manders coefficients A vs. B based on the dual-color DNA-PAINT images. The experiment was performed in triplicate (N = 3) and included seven cells overall with 29 analyzed ROIs. (**C**) Violin plots of Manders coefficients for the associations of KRas-G12D or HRas-G12V with PS (A) and PS with either KRas-G12D or HRas-G12V (B). Experiments performed in triplicate (N = 3) including eight and seven cells for each KRas-G12D and HRas-G12V, with 55 and 29 analyzed ROI, respectively, were used for this graph.

Figure 3A shows a representative image and zoom-ins of the molecular distribution of HRas-G12V (red) and PS (green). As in the case of KRas-G12D, stably expressed HRas-G12V was universally detected across the plasma membranes of all cells, and only a fraction of those were transiently transfected with Lact-C2-GFP (Figure 3A). In the GFP-positive cells, Lact-C2 localizations appeared to be largely uniform across the whole membrane except for regions where the membrane seemed to ruffle or form protrusions, resembling those observed on the KRas-G12D or blank cells (compare Figure 1D, Figure 2B, and Figure 3A). The distributions of Lact-C2 were visually similar in the zoom-in views for all three cases (Figure 3A(a–c)). Despite this similarity, however, a more systematic investigation is needed before concluding that KRas-G12D or HRas-G12V expression (at the levels seen in this work) did not alter the overall distribution of PS on U2OS cell membranes. 

To quantify the spatial relationship between HRas-G12V and PS, we again performed Coloc-Tesseler colocalization analysis of multiple (1000 pixels^2^ area on average) ROIs in a number of Lact-C2-GFP positive cells. Somewhat surprisingly, the Manders A coefficients (HRas-G12V overlapping with PS) were almost always close to 1, suggesting extensive co-enrichment of HRas-G12V with PS (Figure 3B). We reiterate that Coloc-Tessler analyzes target co-enrichment, i.e., where both targets are of high density. Thus, a Manders A coefficient of 1 implies that target A is always enriched where the other target (target B) is enriched. As expected, the opposite is not the case. Judging from the Manders B coefficient, there was a wide-range of variability, from ~0 to 0.8, among the ROIs in the percentage of PS enriched regions where HRas-G12V was also enriched. 

To better compare the spatial associations between PS and the two Ras isoforms, we plotted all the Manders A and B coefficients in a violin plot in Figure 3C. The Manders A coefficients for KRas-G12D over PS clearly exhibit a bimodal distribution centered around ~0.95 and ~0.4 (±0.2), respectively, with an approximately 1:1 split ratio between the two populations. As noted earlier, this split is between cells, not ROIs. In contrast, nearly all of the ROIs (and cells) from HRas-G12V-expressing cells showed HRas-G12V co-enriching with PS. The distributions of Manders B coefficients are more similar, although the degree of variation in the HRas-G12V-expressing cells appeared to be somewhat larger compared with that in the KRas-G12D-expressing cells. These results suggest that while PS is spatially associated with both mutant Ras isoforms, there is a clear difference in their association patterns. 

### 3.4. Dependence of Ras-PS Association on the Actin Cytoskeleton

We next sought to perturb the membrane distribution of PS to better understand its spatial association with Ras. Many approaches have been used to manipulate PS in the membrane, such as inhibition of PS synthesis or trafficking; however, depletion of PS often leads to detachment of Ras from the membrane. Therefore, we chose to apply an indirect strategy by perturbing the actin cytoskeleton. Dynamic actin polymerization has been linked to PS mobility and spatial organization [22,26,46,47], and the spatial clustering of Ras isoforms have been shown to differentially depend on the actin cytoskeleton [9].

In this work, we used two common actin perturbing agents, Jasplakinolide (Jas) and latrunculin A (LA), to modulate actin dynamics and assess their impacts on Ras–PS associations. Short dynamic actin filaments are key to PS immobilization and clustering on the membrane [22,47]. Jas enhances actin nucleation, leading to stabilization of short actin oligomers, although its exact effect on PS dynamics is somewhat ambiguous [26,48]. LA, on the contrary, binds to and sequesters actin monomers, thus preventing them from being added to the filament end of F-actin [49].

**Figure 4:**
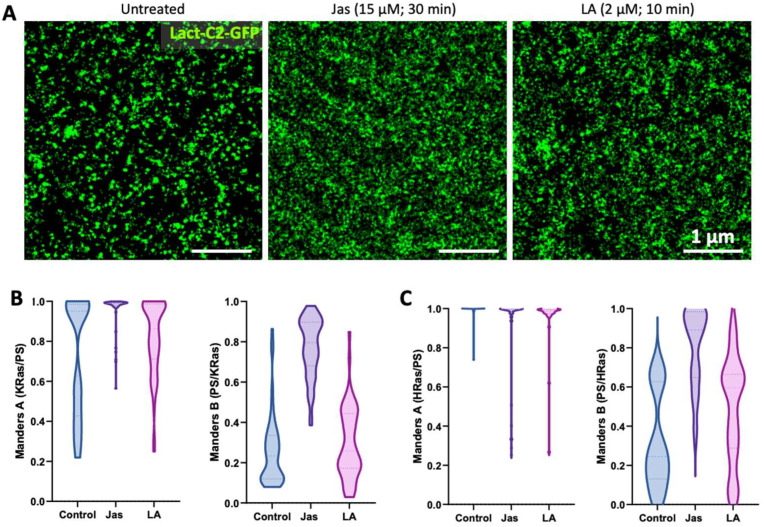
Assessment of the role of the actin cytoskeleton in Ras-PS interactions through DNA-PAINT imaging. (**A**) Impact of actin perturbations on the spatial distribution of PS on the plasma membrane of U2OS cells. Example 4 µm × 4 µm areas of DNA-PAINT images taken on U2OS cells transiently transfected with Lact-C2-GFP and treated as indicated. (**B**) U2OS cells stably expressing SNAP-KRas-G12D were transfected with Lact-C2-GFP and treated with a vehicle (15 μM Jasplakinolide (Jas) for 30 min or 2 μM Latrunculin A (LA) for 10 min) before imaging. Molecule localizations were analyzed with Coloc Tesseler and violin plots were prepared showing Manders A (left) and Manders B (right) coefficients for the treated or untreated cells. (**C**) U2OS cells stably expressing Halo-HRas-G12V were treated similarly; the violin plots show Manders A (left) and Manders B (right) coefficients. Control conditions included eight and seven cells for both KRas-G12D and HRas-G12V expressing cells, and experiments were performed in triplicate (N = 3) with 55 and 29 ROIs analyzed, respectively. The Jas treatment group included seven KRas and six HRas cells, and was performed in duplicate (N = 2) with 52 and 44 ROIs analyzed, respectively. The LA group included eight KRas and six HRas cells, and was performed in two independent experiments with 54 and 34 ROIs analyzed, respectively.

**Figure A4:**
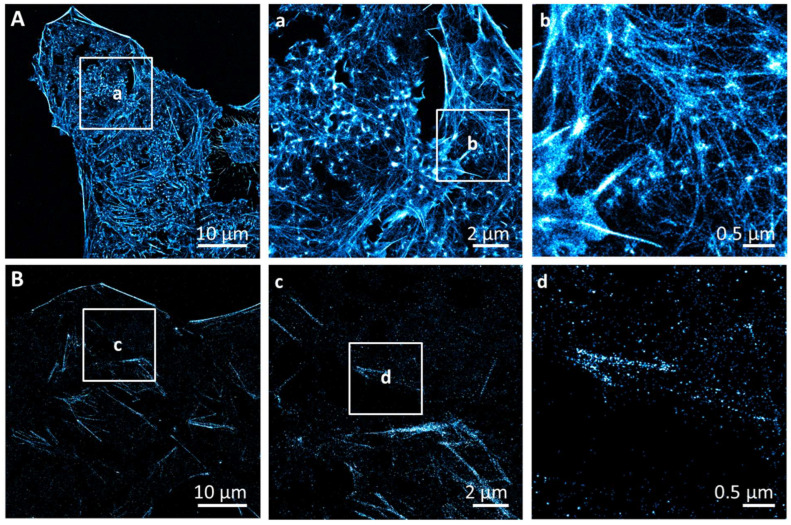
STORM imaging of U2OS cells, showing the effect of actin depolymerization. (**A**) U2OS untreated control cells were fixed with 3.7% PFA, stained with Alexa Fluor™ 647 Phalloidin (#A22287), and imaged in a standard STORM imaging buffer with 100mM MEA, as previously described [50,51]. Magnifications (**a**) and (**b**) show intact F-actin fibers. (**B**) U2OS cells treated with 2 μM LA for 10 min stained with Alexa Fluor™ 647 Phalloidin and imaged with STORM. Magnifications (**c**) and (**d**) clearly show a significant reduction in large fibers that are characteristic of depolymerization of the actin cytoskeleton. The experiment was performed twice with similar results (N = 2), and included three cells for each condition.

U2OS cells expressing Lact-C2-GFP and either SNAP-tagged KRas-G12D or Halo-tagged HRas-G12V were treated with either 15 μM Jas for 30 min or 2µM LA for 10 min to promote or inhibit actin polymerization, respectively. Depolymerization of the actin cytoskeleton upon treatment with LA was confirmed using stochastic optical reconstruction microscopy (STORM) imaging after labeling with Alexa Fluor™ 647 Phalloidin [50,51] (Figure A4). STORM images showed that after 10 min of treatment at 2 µM, the majority of the F-actin meshwork and most of the large fibers disappeared, although some of the thick stress fibers and potentially many short filaments remained. As expected, both treatments caused significant changes in cell morphology (data not shown). In the resulting DNA-PAINT images of Lact-C2-GFP, it was apparent that both Jas and LA caused redistribution of PS on the cell membrane. Compared with the distinct puncta of PS in control cells (Figure 4A, left; see also Figure 1D, Figure 2D, and Figure 3A), PS in the treated cells appeared more diffusive. In all cases, however, PS distributions remained nonrandom, with apparent clusters of various sizes persisting even after treatment. 

Actin stabilization with Jas had a much stronger effect on the spatial association be-tween KRas-G12D with PS than HRas-G12V. Jas treatment shifted the Manders A coefficient for KRas-G12D and PS from 73% towards 96% (nearly complete association) for all cells analyzed (Figure 4B). The Manders B coefficient significantly increased from 25% to 77% after Jas treatment showing higher association of PS-enriched regions with KRas-G12D. Figure 4C shows the effect of actin perturbation on HRas-G12V and PS co-organization. Here, the Manders A shows a high co-occurrence of areas that are enriched in HRas-G12V and PS independent of actin cytoskeleton assembly or disassembly. However, the Manders B coefficient increased from 32% to 77% in response to actin polymerization following Jas treatment, indicating a higher co-occurrence of PS in HRas-G12V-enriched areas. 

In comparison, LA treatment had a much smaller effect on Ras–PS associations compared with that observed for Jas. LA treatment led to a less pronounced increase of Manders A from 73% to 82% in KRas-G12D and PS co-organization, whereas the Manders B coefficient for KRas-G12D was unaffected by LA treatment. Disassembling actin with LA treatment had no effect on the colocalization of HRas-G12V with PS and an intermediate effect on the association of PS with HRas-G12V (49%). Manders B is higher for cells expressing HRas-G12V compared to KRas-G12D, which is an indication that PS prefers co-organization with HRas-G12V compared to KRas-G12D. 

## 4. Discussion

While the membrane plays a critical role in regulating Ras activity, how Ras interacts with various membrane compartments remains unclear. In this work, we used dual-color DNA-PAINT(ERS) super resolution imaging to map PS and Ras (HRas-G12V or KRas-G12D) at the nanoscale on the native plasma membrane (Figure 1). By combining three miniature probes, namely, an anti-GFP-nanobody, the SNAP-tag (for labeling of KRas-G12D), and the Halo-tag (for labeling HRas-G12V), we were able to achieve efficient labeling and high-resolution imaging of these species in cells. Our results confirm previous observations from immuno-EM imaging of membrane sheets [23,30] and computational studies [12,13,14,15,16,17,18], and offer new insights into the spatial relationship between Ras and PS. 

Using exchange-PAINT, we confirmed that KRas-G12D co-enriches with PS on the cell membrane (Figure 2). Interestingly, by imaging KRas and PS in many cells, we observed two cell populations that showed either moderate or strong co-enrichment of KRas-G12D with PS (Figure 2H). The variation is inter-cellular (cell-to-cell) and not intracellular (different ROIs within the same cell), indicating that the variation may be correlated with the biological state of each cell, although at present the cause of this variation is unclear. Among the possibilities, KRas phosphorylation [52,53] or other types of post-translational modifications could impact its association with PS across the whole cell membrane. In addition, certain scaffold mechanisms such as the actin cytoskeleton may directly impact the extent of KRas–PS association. Indeed, treatment with Jas altered both the spatial distribution of PS and significantly increased the level of co-enrichment of KRas with PS. More experiments are clearly needed to define the two cell populations as well as the mechanisms through which actin perturbation with Jas enhances KRas association with PS.

PS was identified as a component of the nanoclusters of both KRas and HRas in previous work [24], although our observation that HRas-G12V exhibited an even more persistent spatial association with PS than KRas-G12D is still somewhat surprising. Aside from the well-known electrostatic interaction with the negatively charged PS, the C-terminal tail (HVR) of KRas has been shown to specifically recognize PS [28,54]. Neither mechanism exists for HRas to strongly associate with PS. Furthermore, the association of HRas-G12V with PS is resistant to perturbations to the actin cytoskeleton, including both Jas and LA treatments. This implies that HRas may spatially associate with PS through mechanisms that are distinct from those mediating KRas and PS interactions, which may account for their different sensitivity to perturbations. Of note, KRas is associated with PS with certain acyl chain compositions [28], and it is plausible that HRas may exhibit distinct selectivity when interacting with PS. 

The actin cytoskeleton facilitates diverse cellular processes and is highly involved in the spatiotemporal organization of the membrane by providing a barrier that restricts diffusion of proteins and lipids [55]. PS directly interacts with actin-binding proteins, and actin association mediates immobilization of PS yielding PS-enriched membrane patches that can potentially entrap membrane proteins such as Ras [56,57]. The observation that actin depolymerization had a limited effect on the spatial association of either Ras form with PS might be due to residual fine actin filaments that persisted after LA treatment (Figure A4), or to the presence of alternative mechanisms to the actin cytoskeleton that might co-enrich Ras and PS. Future work should involve incorporation of actin into the multiplexed imaging palate of DNA-PAINT in order to investigate the involvement of actin in the spatial regulation of Ras and PS on the membrane. 

Aside from PS, other anionic lipids such as PIP2 have been found to interact with Ras, although current studies have not reached a consensus as to whether and to what extent PIP2 (and other PI lipids) is important in the spatial organization and function of Ras [13,14,15,58]. Ras function involves multimer formation and interaction with other proteins, such as scaffolds and downstream effectors. A comprehensive understanding of how Ras forms multimers and functions on the membrane requires tools that could map many of the molecular players on intact membranes at once. With advances in multiplexed super resolution microscopy and the development of novel affinity agents, we expect that to become a possibility in the near future.

## Figures and Tables

**Table 1:** Primer design for anti-GFP nanobody production.

Primer:	DNA Sequence
Anti-GFP-Nb-F	GAAGGAGATATACATATGGCGCAGGTTCAGCTGGTTGAAAGC
Anti-GFP-Nb-R	TGCGGCCGCAAGCTTCTAGCTGCTAACGGTAACCTGGGTG
pET21b-F	AAGCTTGCGGCCGCACTCGAG
pET21-R	ATGTATATCTCCTTCTTAAAGTTAAACAAAATTATTTCTAGAGGGGAATTGTTATCCGC

## Data Availability

All relevant data have already been included in this manuscript. Raw DNA-PAINT images and noncurated analysis results are available upon request.
